# Molecular evolution of cyclin proteins in animals and fungi

**DOI:** 10.1186/1471-2148-11-224

**Published:** 2011-07-28

**Authors:** Konstantin V Gunbin, Valentin V Suslov, Igor I Turnaev, Dmitry A Afonnikov, Nikolay A Kolchanov

**Affiliations:** 1 Institute of Cytology and Genetics, Siberian Branch of the Russian Academy of Sciences, Lavrentyev ave., 10, Novosibirsk, Russia; 2 Novosibirsk state University, Pirogova, 2, Novosibirsk, Russia

## Abstract

**Background:**

The passage through the cell cycle is controlled by complexes of cyclins, the regulatory units, with cyclin-dependent kinases, the catalytic units. It is also known that cyclins form several families, which differ considerably in primary structure from one eukaryotic organism to another. Despite these lines of evidence, the relationship between the evolution of cyclins and their function is an open issue. Here we present the results of our study on the molecular evolution of A-, B-, D-, E-type cyclin proteins in animals and fungi.

**Results:**

We constructed phylogenetic trees for these proteins, their ancestral sequences and analyzed patterns of amino acid replacements. The analysis of infrequently fixed atypical amino acid replacements in cyclins evidenced that accelerated evolution proceeded predominantly during paralog duplication or after it in animals and fungi and that it was related to aromorphic changes in animals. It was shown also that evolutionary flexibility of cyclin function may be provided by consequential reorganization of regions on protein surface remote from CDK binding sites in animal and fungal cyclins and by functional differentiation of paralogous cyclins formed in animal evolution.

**Conclusions:**

The results suggested that changes in the number and/or nature of cyclin-binding proteins may underlie the evolutionary role of the alterations in the molecular structure of cyclins and their involvement in diverse molecular-genetic events.

## Background

The progression through the cell cycle (the G1→S→G2→M transition) is mainly controlled by the enzymic activities of cyclin-dependent kinases (CDKs). The association of cyclins with CDKs is the condition requisite for their activation [1-3]. Cyclins have been discovered in sea urchin eggs as proteins whose synthesis and degradation oscillate during the cell cycle [4]. Periodic changes in the concentration of cyclins cause sequential activation/inactivation of the CDK catalytic partners resulting in periodic advancement of cells through the cell cycle [2,5,6]. Most metazoans have four cyclin types A, B, D and E, which regulate cell-cycle transitions. For example, cyclin D controls the G1 phase by interacting with CDK4 and CDK6; cyclin E interacts with CDK2 and controls the end of the G1 phase and the transition through G1/S; the cyclin A/CDK2 complex regulates the S phase and the exit from it and the cyclin A/CDC2(CDK1) complex regulates the G2/M transition; cyclin B interacts with CDC2 and also regulates the G2/M transition. The major cyclins are those that directly regulate the cell cycle, when in complex with CDKs. The others, which perform auxiliary functions, are united into the group of additional cyclins [7]. In fungi, of the four major cyclin families characteristic of animals, only B-type cyclins are present. In *Schizosaccharomyces pombe*, there is just one family of these major cyclins, CDC13. In *S. pombe *Cig1 and Cig2 null-mutants, Cdc13 can alone provide orderly progression through cell cycle [8]. In the other fungi, such as *Saccharomyces cerevisiae*, there are several cyclins of B family (CLB1-2, CLB3-4, CLB5-6), which control the different phases of the cell cycle (CLB5-6, S phase; CLB1-2, CLB3-4, G2 and M phases) [6].

Cyclins are highly conserved proteins identified in fungi, plants, animals and protists [9,10]. In recent years, based on genome-wide and comparative phylogenetic analyses, numerous studies have been conducted to define cyclin relatedness. Thus, 49 cyclins, assigned to 10 families, were identified in the *Arabidopsis thaliana *genome [11], 49 cyclins forming 9 families were detected in the *Oryza sativa *genome [12], the number of cyclins, the members of 6 families, was determined as 59 in *Zea mays *[13]. The relatedness of cyclins in the unicellular green algae *Ostreococcus tauri *[14], and diatom algae *Thalassiosira pseudonana *and *Phaeodactylum tricornutum *[15] has been examined. Furthermore, using genome-wide data, the relationship of distinct cyclin families was studied: cyclins of D-type in the Angiosperms (*Arabidopsis thaliana, Oryza sativa, Zea mays, Populus alba*) and the Bryophytes (*Physcomitrella patens*) [16] and cyclins of A-type [17], B-type [17,18], D-type [18,19] and E-type [18] in animals and fungi. On the basis of phylogenetic tree analysis, the relationships among cyclins A, B, D and E in different animal taxa were investigated [20]. In these recent studies, a particular focus has been on assignments of cyclin sequences to subfamilies. However, detailed analysis of phylogeny and evolution modes of proteins (in this context, by evolution modes, we mean a statistically significant acceleration or deceleration of accumulation of amino acid replacements) would be useful not only in a reconciliation of classification issues, it would also enable us to identify, with more reasonable accuracy, protein function features. The narrow taxonomic diversity of sequenced plant species and their polyploidy makes their statistical analysis very difficult. For this reason, plants were disregarded. With this stipulation, here we analyze the phylogeny and evolution modes of distinct cyclin families belonging to animals and fungi.

Over the past decade, the relation between the level of the expression of the gene and its evolutionary rate generated great interest. In 2005-2009, it was shown for the first time that this relation is universal, resulting from the selection against the toxic effect of protein misfolding. Protein misfolding may possibly be induced by (1) translation errors; (2) misfolding during erroneous translation; (3) spontaneous misfolding and unfolding [21-23]. All force selection applies to counteract protein misfolding, which is obviously due to amino acid replacements, affecting differently the protein structure and function. Hence, a promising study of changes in molecular functions of proteins and protein encoding genes during evolution would place emphasis on changes in accumulation rates of amino acid replacements, and their localization in protein 3D structure, thereby providing a better understanding how amino acid replacements became fixed during evolution.

In the current study, we examine the structural-functional features of the molecular evolution of the of A-, B-, D-, E-type cyclin proteins of animals and fungi. An analysis of the fixation of amino acid replacements atypical of the four cyclin families disclosed the evolutionary features of structural cyclin regions, where these replacements occurred. The results made it possible to relate the evolutionary features of cyclins with trends of the ecological specialization apparent in the course of evolution of fungi, vertebrates and invertebrates. We discerned a broad pattern, encompassing virtually all the atypical amino acid replacements, the more superficial relative to protein surface (the surface replacements) became more frequently fixed during evolution, whereas those further away from the surface (the interior replacements) were rarely, if at all, fixed during it. Altered protein-protein interactions, and changes in the number and/or nature of cyclin-binding proteins, in particular, possibly underlay the evolutionary relationship between changes in molecular structure of cyclins and their involvement in the molecular-genetic events in distinct taxa.

## Methods

### Species studied

Here, we analyze a total number of 54 species representing animals and fungi whose genomes have been completely sequenced. Protist and placozoan cyclins from 7 fully sequenced species served as outgroups in phylogenetic analysis. In so doing, we minimized the consequences of possible insufficient data on cyclin sequences for any organism. The following taxonomic groups of organisms were included in analysis (Table 1): Mammalia; Aves; Amphibia; Actinopterygii; Tunicata; Cephalochordata; Echinodermata; Insecta; Nematoda; Cnidaria; Ascomycota; Basidiomycota. The following taxonomic groups of organisms served only as outgroups in our analysis (Table 1): Placozoa; Choanoflagellida; Amoebozoa; Euglenozoa; Diplomonadida.

**Table 1:** Species studied and number of their cyclins analyzed

Phylum	Class	Species	Number of cyclins analyzed			
			
			A	B	D	E
*Kingdom Animalia*						

Chordata	Mammalia	*Bos taurus*	2	2	3	2
		*Canis familiaris*	2	2	2	2
		*Equus caballus*	2	3	2	2
		*Homo sapiens*	2	3	1	2
		*Macaca mulatta*	2	1	1	1
		*Monodelphis domestica*	2	2	2	2
		*Mus musculus*	2	3	3	2
		*Ornithorhynchus anatinus*	2	1	0	1
		*Rattus norvegicus*	2	2	3	2
		*Sus scrofa*	1	1	2	0
	
	Aves	*Gallus gallus*	2	2	3	2
		*Taeniopygia guttata*	2	1	3	2
	
	Amphibia	*Xenopus laevis*	2	2	2	2
	
	Actinopterygii	*Danio rerio*	2	3	1	2
		*Oryzias latipes*	1	3	2	2
		*Takifugu rubripes *syn. *Fugu rubripes*	1	1	1	2
		*Tetraodon nigroviridis*	1	2	2	1
	
	Ascidiacea	*Ciona intestinalis*	1	2	0	1
	
	Cephalochordata	*Branchiostoma floridae*	1	1	1	1

Echinodermata	Echinoidea	*Strongylocentrotus purpuratus*	1	2	1	1

Arthropoda	Insecta	*Acyrthosiphon pisum*	1	0	2	0
		*Aedes aegypti*	1	2	0	1
		*Anopheles gambiae*	1	2	0	0
		*Apis mellifera*	1	2	1	1
		*Culex quinquefasciatus *syn. *Culex pipiens quinquefasciatus*	0	2	0	1
		*Drosophila grimshawi*	1	0	1	1
		*Drosophila melanogaster*	1	2	1	1
		*Drosophila mojavensis*	0	1	0	0
		*Nasonia vitripennis*	1	2	0	1
		*Tribolium castaneum*	1	1	2	1

Nematoda	Secernentea	*Brugia malayi*	1	0	0	0
		*Caenorhabditis elegans*	1	2	1	1

Cnidaria	Hydrozoa	*Hydra magnipapillata*	1	2	1	1
	
	Anthozoa	*Nematostella vectensis*	1	2	1	1

Placozoa	Tricoplacia	*Trichoplax adhaerens*	1	1	0	1

Total cyclin number analyzed in kingdom Animalia			46	60	45	43

*Kingdom Fungi*						

Ascomycota	Saccharomycetes	*Ashbya gossypii*	-	3	-	-
		*Candida albicans*	-	2	-	-
		*Candida glabrata*	-	1	-	-
		*Yarrowia lipolytica*	-	1	-	-
		*Vanderwaltozyma polyspora*	-	3	-	-
		*Kluyveromyces lactis*	-	3	-	-
		*Pichia stipitis*	-	2	-	-
		*Saccharomyces cerevisiae*	-	3	-	-
		*Debaromyces hansenii *syn. *Debaryomyces hansenii*	-	2	-	-
	
	Eurotiomycetes	*Aspergillus nidulans*	-	1	-	-
		*Neosartorya fischeri*	-	2	-	-
	
	Leotiomycetes	*Botrytis cinerea *syn. *Botryotinia fuckeliana*	-	1	-	-
	
	Sordariomycetes	*Fusarium graminearum*	-	1	-	-
		*Magnaporthe grisea*	-	1	-	-
		*Neurospora crassa*	-	1	-	-
		*Podospora anserina*	-	1	-	-
	
	Schizosaccharo-mycetes	*Schizosaccharomyces pombe*	-	1	-	-
	
	Leotiomycetes	*Sclerotinia sclerotiorum*	-	1	-	-

Basidiomycota	Tremellomycetes	*Cryptococcus neoformans*	-	1	-	-
	
	Exobasidiomycetes	*Malassezia globosa*	-	1	-	-

Total cyclin number analyzed in kingdom Fungi			-	32	-	-

*Kingdom Protista*						

Choanozoa	Choanoflagellatea	*Monosiga brevicollis*	1	1	-	-

Amoebozoa syn.	Dictyostelia	*Dictyostelium discoideum*	0	1	-	-
	
Mycetozoa	Archamoebae	*Entamoeba dispar*	0	1	-	-

Euglenozoa	Kinetoplastida	*Leishmania major*	0	1	-	-
		*Trypanosoma cruzi*	0	1	-	-

Metamonada	Diplomonadida	*Giardia lamblia*	0	1	-	-

Total cyclin number analyzed in kingdom Protista			1	6	-	-

### Orthologous groups of cyclins

The protein sequences we analyzed were retrieved from the KEGG 52.0 database [24]. Assignments of animal and fungal cyclins to orthologous groups were extracted from KEGG Orthology [24,25]. The protein orthologous groups were as follows: K12760 (CLN1), K06650 (CLN2), K06646 (CLN3), K11115 (CDC13), K02220 (CLB1-2, CIG2), K06659 (CLB3-4), K06651 (CLB5-6), fungi and protists; K04503 (CCND1), K10151 (CCND2, CycD), K10152 (CCND3), K06626 (CCNE), K05868 (CCNB), K06627 (CCNA), animals and protists. Based on KEGG Orthology, protein sequences for each of the above listed groups of orthologs were taken from the KEGG Genes database [24,25]. Then, to enrich the sample, five proteins, which were most similar to the query protein by primary structure, were extracted from the KEGG SSDB database. Duplicate sequences and/or identical protein sequences were discarded. The resultant sample contained 233 of A-, B-, D-, E-type cyclin proteins.

### Multiple alignment, reconstruction of phylogeny and ancestral sequences

A PROMALS3D web server was used for the multiple alignment of amino acid sequences using protein structure data [26]. The primary alignment was done using data on the secondary and tertiary structure of cyclins [27]. The obtained alignment was divided into 4 secondary alignments for each cyclin type, A, B, D and E (Additional file 1).

It is well known that the accuracy of phylogenetic reconstruction, i.e., the precision of estimated branch lengths, considerably increases when models of relative replacement rates of amino acids, specific to a particular protein family, are examined [28,29]. With this in mind, we built models for relative amino acid replacement rates using gapless secondary alignments by MODELESTIMATOR 1.1 for each cyclin family [30]. Then the phylogenetic trees for cyclins of A-, B-, D-, E-type were reconstructed using these models and gapless secondary alignments by PhyML 3.0 [31].

Phylogenetic trees derived from gapless secondary alignments, were used for the analysis of phylogenetic relationships. Support values for each node of the phylogenetic tree from the gapless secondary alignment was provided by the approximate likelihood-ratio test for branches [32] and the test, based on multiple comparisons of log-likelihoods elaborated by Shimodaira and Hasegava [33]. Because cyclins are an evolutionary conserved group of proteins, the trees derived from their sequences were multifurcated. In such confounding instances, the issue was resolved manually, relying on the literary data and on the internet resource Tree of Life [34]. To define the divergence order of the unicellular ancestor of multicellular animals and fungi, we took advantage of genome-wide data [35-38]. The issue of phylogenetic relationships within fungal taxa was also resolved on the basis of genome-scale data [38-40]. To define tree topology at the divergence level of nematodes, arthropods and deuterostomes, we had recourse to evidence in favor of the existence of the ecdysozoans [41-48]. In the construction of the tree at the level of the divergence of echinodermates, cephalochordates, chordates and tunicates, additional information was used [43,49-51]. The divergence order of organisms within a mammalian class was verified [52-56], and so was that within an arthropod group [57]. The divergence order of paralogous cyclins in fungi, the group most susceptible to the effects of heterotachy during evolution [58], was consistent with that reported earlier [59].

The phylogenetic trees with multifurcations resolved as described above were utilized for the accurate estimation of branch lengths and the gamma shape parameter α using gapless secondary alignments by PhyML 3.0 [31]. The above estimates and gapless secondary alignments, were used to reconstruct ancestral sequences in tree inner nodes for each of the A-, B-, D-, E-type cyclins by the maximum-likelihood method. Three algorithms were applied for the reconstruction of ancestors differing by their approach to the optimization of ancestral protein reconstruction: 1) ANCESCON [60] used the general WAG model for relative amino acid replacement rates [61]); 2) FASTML server [62] took into account the general LG model for relative amino acid replacement rates [63]; 3) the specific models for the relative amino acid replacement rates concerning each cyclin family, which we constructed by MODELESTIMATOR 1.1 [30], were used in the ancestral reconstruction by AAML software (the PAML package 4.3b, the CODEML program [64]). In the reconstruction of the ancestor, we used only the marginal approach because it is the one that enables us to reconstruct the most probable protein sequences for each inner node of phylogenetic tree [65]. Having reconstructed the ancestral sequences in all the inner nodes of the four cyclin phylogenetic trees by all the three algorithms (Additional file 2; Additional file 3), we reconstructed three common ancestors of the cyclin families by the ANCESCON program [60]: the ancestor of A and B, and the ancestor of D and E, the ancestor common to all the four cyclin families for each of the three methods of reconstruction.

### Search of atypical amino acid replacements in sequences in the phylogenetic tree branches

In this analysis, a replacement of an amino acid *a *(or *b*) in an ancestral node by an amino acid *b *(or *a*) in a descendant node was designated as *atypical*, if the occurrence probability of the *a→b *replacements was small. Such replacements may reflect important changes in the protein function fixed during evolution; however, they may indicate the increasing rate of amino acid substitutions in a particular branch. To identify atypical amino acid replacements in sequences corresponding to each branch, we compared the number of all the observed 190 types of amino acid replacements with what was expected, accepting that cyclin evolution is a stationary homogeneous Markov process. The number of amino acid replacements observed for each type, *n*(*Type_R_*), was calculated in the inner branches by pairwise comparison of ancestral-descendant sequences reconstructed by three methods: AAML [64], FASTML [62] and ANCESCON [60] (see above). For the terminal nodes, the observed number of amino acid replacements was calculated by pairwise comparison of ancestral sequence, reconstructed also by three different methods, with a descendant sequence taken from an extant organism. In further analysis, we considered the ancestral amino acids whose reconstruction probability was greater than 0.99 (using estimates from each of the reconstruction methods (Additional file 3)).

The expected values for replacements were calculated on the basis of the computer simulation of cyclin molecular evolution with the package INDELible 1.03 [66]. The sequence evolution was simulated on the basis of a phylogenetic tree of A-, B-, D-, E-type cyclins taking into consideration the gapless alignments of these proteins (length; proportion of conserved columns; the gamma shape parameter (α), which describes the heterogeneity of evolutionary reorganization), and the matrix for amino acid replacement rates, estimated for each cyclin family (Additional file 4). Proceeding on the data from these 1000 alignments for each branch (including the branch leading to the terminal nodes), the rates expected for amino acids of 190 types were calculated.

The expected and observed replacements of each type were compared by the permutation test we developed earlier [67]. This type of statistical test [68] allowed us to estimate the deviation and the significance of the observed amino acid replacement types from the expected ones for each branch. A set of observed replacements was always included in the expected set. Thus, for a simulated set of replacements, 10^5 ^random samples of the same size as those containing observed replacements were generated by random permutation. The number of expected amino acid replacements *nrand*(*Type_Rrand_*), which belonged to a given type, 190 in all, was estimated for each random sample. Also the number of random samples *M *in which *nrand*(*Type_Rrand_*) >*n*(*Type_R_*) was estimated in the course of the test. Therefore, the *M*/10^5 ^value expressed the probability *p *at which the occurrence of the observed replacements of each type (*Type_R_*) could have arisen by chance (Additional file 5). The strict threshold *p *≤ 0.01 was used to define the atypical amino acid replacements (the less probable according to the stationary and homogeneous Markov model). In our analysis, we considered only tree branches with atypical replacements (*p *≤ 0.01) identified using all the three reconstruction methods (AAML, FASTML and ANCESCON).

### Estimation of accessibility of protein to solvent, prediction of secondary structure

The accessibility of amino acids to solvent and their reference to particular regions of secondary structure were determined in protein 3D structure applying the DSSP program [69]. Representative proteins with known 3D structures for the A, B1/B2, E and D cyclin families we taken from the PDB database [70]: 1H1R:B (KEGG ID 890) for cyclin A family; 2W9Z (KEGG ID 595) for cyclin D family; 2B9R:A (KEGG ID 891) for cyclins of B1, B2 families; 1W98:B (KEGG ID 898) for cyclins of E family. 3D structures of the representative proteins of B3 and fungal B cyclin families were reconstructed by Phyre server [71] using 2B9R:A as template structure: 3D model of *Homo sapiens *cyclin B3 (KEGG ID 898); 3D model of the *Saccharomyces cerevisiae *cyclin B (KEGG ID YGR108W). Protein tertiary structure alignment was performed by the CE program [72].

In determination of the relation between the accessible surface area (ASA) with atypical amino acid replacement types, the true *ASA_i _*values of residue *i*, obtained with the DSSP program [69], also 10 classes (*C *= 10(*ASA_i_*/*ASA_max_*) of relative ASA values were used. To scale the true ASA values, the ASA of the extended states of Ala-X-Ala for every residue × (*ASA_max_*) are used under the assumption that the absolute values include side chain and backbone surface area [73]. These values are (in Å^2^) 110.2 (Ala), 144.1 (Asp),140.4 (Cys), 174.7 (Glu), 200.7 (Phe), 78.7 (Gly), 181.9 (His), 185.0 (Ile), 205.7 (Lys), 183.1 (Leu), 200.1 (Met),146.4 (Asn), 141.9 (Pro), 178.6 (Gln), 229.0 (Arg), 117.2 (Ser), 138.7 (Thr), 153.7 (Val), 240.5 (Trp), and 213.7 (Tyr), respectively [73]. The relative ASA values were roughly assigned to three categories: amino acids belonging to the *ASA *classes *C *≤ 2 were considered as buried (internal), those belonging to the classes 2 <*C *≤ 4 as subsurface (intermediate), and those belonging to *C *> 4 as surface (external).

### Comparison of the number of expected and observed replacements for each secondary structure

The number of expected replacements was estimated for each secondary structure element (for example, each α-helix or the β-strand) of protein of animal cyclin A, D and E families and for animal cyclin B, fungal cyclin B families and animal B3-cyclins subfamilies. For this purpose, the total number of amino acid replacements and their number in each structure element observed from the roots to the tips of the trees for animal A-, D-, B-, E-, B3- and fungal B cyclins were calculated (Additional file 6). The estimate of the number of expected replacements in the structure element (*e*) was obtained under the assumption that fixed replacements were distributed evenly in the protein sequence: *e = o *(*l/L*), where *l *is the number of residues in the secondary structure element, *L *is the protein length, and *o *is the total number of observed replacements in protein. The significance of the relationship between the frequency of replacements and their location in the secondary structure element was estimated using the χ^2 ^test with *k*-1 degrees of freedom, where *k *is the number of secondary structure elements. The choice of the secondary structures whose number of observed replacements was significantly smaller or greater than expected, was based on a comparison of the *r *= (*e -- o*)^2^/*e *values with the critical χ^2 ^value for probability *p ≤ *0.01 at given degrees of freedom (Additional file 6).

Those secondary structure elements of animal A-, D-, B-, E-, and B3- and fungal B- type cyclins, whose number of observed replacements significantly differed from the expected (*p *≤ 0.01) in all three ancestral reconstructions (AAML, FASTML and ANCESCON), were included in the final analysis.

### Analysis of the evolutionary rates distribution

To analyze the variation in the rates of amino acid replacements within the cyclin families, subtree branch length distributions were examined in terms of their ranks. Comparisons based on the branch rank values allowed us to disregard the possible deviation of the branch length distribution from the Gaussian shape. The procedure for the examination of branch length distribution for the subtrees was multistep. First, the length of branches for every of the four cyclin family tree from root to tips were measured. Then, with the R:Stats program package [74], rank values were assigned to every branch of the cyclin family trees. At the last step, for all the analyzed subtrees of a cyclin family tree, *R_m_*, the rank median for subtree rank length and *R_v_*, the difference between the maximum and minimum rank lengths of the subtree divided by branch number in the subtree, were calculated. *R_m _*described the branch length in the analyzed subtree, relative to the lengths of all the branches in the cyclin family tree, while *R_v _*expressed the variation in the length of the subtree branches. Obviously, the greater was the subtree *R_m_*, the faster the protein evolved within the cyclin family tree and the smaller was the subtree *R_v _*(absolute minimum *R_v _*= 1), the closer were the evolution rates within the subtree.

## Results and Discussion

### General analysis of evolution rates of different families of cyclin proteins

#### Analysis of animal cyclin evolution

**Figure 1:**
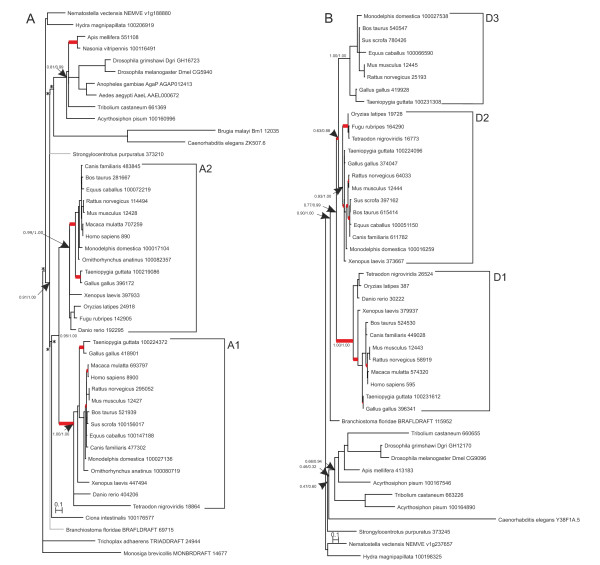
**Phylogenetic trees for cyclins A (A) and D (B)**. Branching significance estimated in calculation of phylogenetic tree was supported by numerical values (the Shimodaira and Hasegava test and the *a*LRT χ^2 ^test). Bifurcations resolved manually on the basis of literary data are indicated by asterisks (*). Inner branches for which atypical replacements were observed are highlighted in red.

**Figure 2:**
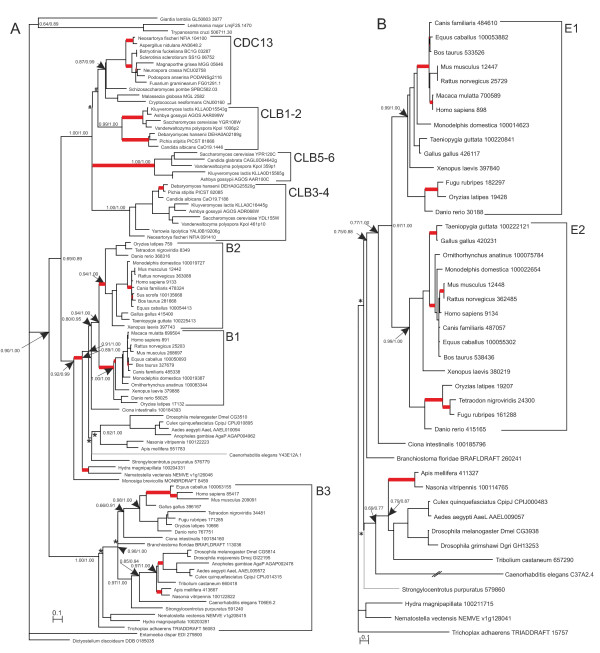
**Phylogenetic tree for cyclin B (A) and cyclin D (B)**. The designations are as in Figure 1. The bifurcations resolved manually on the basis of the data in the literature on fungi are indicated by hatches (#).

Based on the analysis of the branch length distribution within the phylogenetic trees of cyclins A, B, D and E, multicellular animals were divided into two monophyletic groups. These two were represented by two clades of the Metazoa phylogenetic tree, passing from the basal Coelenterates group. Invertebrates, Insects and Echinodermates (with the exception of the Nematodes) were referred to the first group *Inv. Inv *was characterized by long branches in A, B and D cyclin family trees (Figure 1; Figure 2; Table 2) and their wide variations (Table 2). This may be taken to mean that the cyclins of this group evolved both rapidly and at uneven rates. Cyclins resulting from duplications in vertebrates with the formation of a paralogous protein group were referred to the second *Ver *group (Figure 1; Figure 2). *Ver *was characterized by relatively short branch lengths in A, B and D cyclin family trees and the narrowest variation in their lengths (Table 2). It is of interest that *R_m _*and *R_v _*for branch lengths in the B3 subtree of the *Ver *group are close to those in B and A cyclins in the *Inv *group (Figure 1A; Figure 2A; Table 2). Thus, on the basis of the cyclin evolution rates, clubbing of two robust *Ver *and *Inv *groups of animal cyclins may be distinguished (irrespective of the inclusion of Coelenterates into the ingroup). As a result, these two groups of cyclins subdivided the animal species into two simultaneously phyletic and ecocentric groups. Vertebrates (the *Ver *group) were, as a rule, eurybiotic as adults. During their relatively long life, they could withstand drastic changes in the environment and gave little or no preference to particular food [75-77]. This makes it possible for adult vertebrates to easily pass from one habitat to another, and to migrate widely [75-77]. The situation was quite different for invertebrates, the *Inv *group. Their larvae and adults were, as a rule, adapted to specific environmental factors and food, being stenobiotic [76]. Consequently, during evolution, animals either conquered a narrow ecological niche, became specialized (first strategy), or took advantage of a wide range of narrow ecological niches (second strategy), passing from one habitat to another when recourses became inadequate and/or competition heavy [76,78]. The smaller, simply organized animals with short life cycle gave preference to the first strategy. These animals are members of the *Inv *group. In contrast, the mobile, relatively long living animals with their complex organization (*Ver *group) preferred the second strategy [76]. Thus reasoning, it may be explained why cyclins A, B and D evolved slower in vertebrates than in invertebrates.

**Table 2:** Comparison of the branch length and branch length variance for various cyclin groups

Cyclin group	Cyclin subfamily	R_m _for group*	R_v _for group*	No. of terminal nodes in group
*Inv *cyclins: coelenterates, echinodermates, insects.	A	37	3.73	11
	B	44	7.89	9

	B3	78	4.9	11
	D	39.5	3.9	10
	E	9	4	10

*Inv *cyclins: echinodermates, insects.	A	38	4.44	9
	B	64	7.86	7
	B3	83	3.67	9
	D	40.5	3.5	8
	E	10.5	4.75	8

*Ver *cyclins: lower and higher chordates	A1	29	2.59	17
	A2	13	1.18	17
	B1	16	2.69	13
	B2	24	2	15
	B3	75.5	4.9	10
	D1	33	3.36	13
	D2	8.5	1	14
	D3	25	3.44	9
	E1	17.5	2.19	16
	E2	30	2.23	17

*Ver *cyclins: coelenterates, lower and higher chordates	A1	28	2.32	19
	A2	12	1.05	19
	B1	15	2.5	15
	B2	22	1.88	17
	B3	74.5	4.42	12
	D1	28	2.85	15
	D2	8.5	1.38	16
	D3	23	2.82	11
	E1	16.5	2	18
	E2	29	2.05	19

Fungal cyclins	CLB1-2	55	1.57	9
	CLB3-4	68	5.11	7
	CLB5-6	90	1.6	5
	CDC13	41	4.18	11

*R_m _- the branch length characteristic, R_v _- the branch length variance characteristic (see Methods).

The nematodes were exceptions; their cyclins had the longest branches from the root (Figure 1; Figure 2). The evolution of the nematodes had two interesting features: first, mutations of their proteins became fixed at higher frequencies [44,79] and, second, their differentiation was progressive in the sense that cell pluripotency progressively decreased during development [80-84], with the cell division number remaining strictly limited and, hence, the cell number in the adult. The accelerated rate of replacement accumulation in the nematode cyclins may possibly be explained by these features of nematode biology.

#### Analysis of fungal cyclin evolution

Duplication of cyclin B genes and formation of paralogs (CLB3-4; CLB1-2 and CLB5-6) was characteristic of some of the fungal taxa (Saccharomycotina); as for the other taxa, their salient feature was the presence in the genome of just one representative homolog of the cyclin B family, cdc13 (Ascomycetes, Pezizomycotina, the sole representative of Taphrinomycotina, *Schizosaccharomyces pombe*, in the current study, basidiomycetes, a representative of Agaricomycotina, *Cryptococcus neoformans*, and a representative of Ustilaginomycotina, *Malassezia globosa*). In this of interest that, in some Saccharomycotina species, the CLB family was represented by six paralogous genes (CLB3,4; CLB1,2 и CLB5,6), whereas other species lost one of the CLB subfamilies. Of the organisms we analyzed, *Neosartorya fischeri *(Pezizomycotina) had a set of paralogs different from that in the rest of fungi, it also harbored cdc13 and CLB3,4 homologs (Figure 2A). How to account for this diversity of homolog number in the fungal CLB family? One reason of this diversity was perhaps a specific cell cycle regulation that developed in the different taxa under the effect of mycelial organization requiring loss of synchronous nucleus and cell division [85-93], another reason was perhaps other specificity of nuclear-cytoplasmic relationships characteristic of fungi (heterokaryon formation, for example) 85; 87; 94; 95].

#### Punctual and gradual cyclin evolution

Evolution in eukaryotes followed a saltatory and gradual course [96]. In terms of punctual evolution, the gene predominantly accumulated replacements in relatively short timescales. This was accompanied by either gene acquirement of a novel function or by selection pressure relaxation, with evolution nearing neutral. Events of such kind immediately preceded adaptive radiation and, for this reason, they, prevailed in the inner branches [97,98]. Pagel et al. (2006) have suggested a simple test for revealing the prevailing nature of evolution [99]. It should be noted that punctual evolution was associated with dramatic genetic reorganization in the inner branches and evolutionary stasis in the terminal branches. Therefore, during punctual evolution, the relation between the inner node number and total branch length from the root became almost linear (from the power form *n *= *α + βx^δ^*, where *n *is number of nodes, *x *is the phylogenetic path length from root to tip), in this case, there was a statistically significant correlation (*β *> 0, *δ*~1 and *α→*0) [99-101]. In contrast, when evolution was gradual and/or there was a node-density artifact, the relation is not linear, i.e. *β*~0, *δ *> 1 and *α≠*0 [99-101]. It proved that the evolving invertebrate cyclins A and B (*Inv *group), vertebrate cyclins D1 and cyclins B3 (meiosis-specific cyclins [84,102,103]), also fungal cyclins CLB3-4 tended to conform to the punctual pattern (Table 3; *β *> 0, *δ*~1 and *α→*0).

**Table 3:** Statistics for the relation between inner node number and branch length from tree root

Cyclin group	Cyclin sub- families	Pearson correlation; **GLS **(**R:stats **[74]) **fitting of *n *= *α + βx***					**Webster et al**. [101]statistics*; **GLS **(**R:stats **[74]) **fitting of *n *= *βx^δ^***					**Venditti et al**. [100]**and Pagel et al**. [99]statistics**
		
		*R^2^*	*α *± SE	*p*-value	*β *± SE	*p*-value	δ ± SE	*p*-value	*β *± SE	*p*-value	1/δ < 1 & signi-ficant β	Signi-ficant β
*Inv *cyclins: coelenterates, echinodermates, insects.	A	**0.7163**	**0.18 ± 0.12**	**0.1807**	**0.85 ± 0.18**	**0.001**	**1.40 ± 0.36**	**0.0036**	**0.18 ± 0.07**	**0.0308**	**0**	**1**
	B	**0.8456**	**0.44 ± 0.14**	**0.0152**	**0.92 ± 0.15**	**0.0004**	**1.58 ± 0.28**	**0.0007**	**0.31 ± 0.08**	**0.0054**	**0**	**0**
	B3	0.8496	0.92 ± 0.10	0.000008	0.92 ± 0.13	0.00006	2.53 ± 0.38	0.0001	0.68 ± 0.09	0.00004	0	1
	D	0.4892	0.36 ± 0.21	0.1297	0.70 ± 0.25	0.0244	1.79 ± 0.79	0.0537	0.35 ± 0.16	0.0647	0	0
	E	0.3226	0.36 ± 0.24	0.1723	0.57 ± 0.29	0.0868	2.11 ± 1.23	0.1245	0.36 ± 0.18	0.0891	0	0

*Inv *cyclins: echinodermates, insects.	A	**0.5561**	**0.15 ± 0.22**	**0.5080**	**0.75 ± 0.25**	**0.0211**	**1.28 ± 0.51**	**0.0392**	**0.15 ± 0.10**	**0.1810**	**0**	**0**
	B	**0.6706**	**0.47 ± 0.28**	**0.1608**	**0.82 ± 0.26**	**0.0242**	**1.57 ± 0.53**	**0.0311**	**0.31 ± 0.16**	**0.1040**	**0**	**0**
	B3	0.7240	0.97 ± 0.17	0.0007	0.85 ± 0.20	0.0036	2.54 ± 0.65	0.0057	0.68 ± 0.16	0.0036	0	1
	D	0.2700	0.50 ± 0.34	0.1939	0.52 ± 0.35	0.1869	2.30 ± 1.70	0.2240	0.47 ± 0.27	0.1380	0	0
	E	0.0819	0.53 ± 0.48	0.3067	0.29 ± 0.39	0.4920	2.79 ± 3.86	0.4970	0.45 ± 0.43	0.3350	0	0

*Ver *cyclins: lower and higher chordates	A1	0.0380	0.58 ± 0.25	0.0471	0.19 ± 0.33	0.5657	3.73 ± 1.00	0.0020	0.32 ± 0.05	0.00003	0	0
	A2	0.3713	0.38 ± 0.08	0.0014	0.61 ± 0.26	0.0466	3.83 ± 1.19	0.0063	0.37 ± 0.07	0.0002	0	1
	B1	0.1989	1.00 ± 0.06	0	0.45 ± 0.30	01691	11.9 ± 6.36	0.0877	0.89 ± 0.10	0	0	0
	B2	0.8209	0.86 ± 0.04	0	0.91 ± 0.14	0.0001	5.34 ± 0.60	0	0.69 ± 0.04	0	0	1
	B3	**0.6674**	**0.62 ± 0.25**	**0.0372**	**0.82 ± 0.20**	**0.0039**	**1.70 ± 0.47**	**0.0064**	**0.43 ± 0.16**	**0.0257**	**1**	**1**
	D1	**0.9021**	**0.19 ± 0.05**	**0.0046**	**0.95 ± 0.10**	**0.000008**	**1.70 ± 0.17**	**0**	**0.22 ± 0.03**	**0**	**0**	**1**
	D2	0.5802	0.24 ± 0.04	0.0002	0.76 ± 0.22	0.0064	3.33 ± 0.66	0.0003	0.21 ± 0.03	0	0	0
	D3	0.6270	0.24 ± 0.11	0.0614	0.79 ± 0.23	0.0110	2.02 ± 0.65	0.0168	0.23 ± 0.07	0.0162	0	0
	E1	0.4468	0.45 ± 0.14	0.0102	0.67 ± 0.25	0.0245	2.95 ± 0.86	0.0042	0.40 ± 0.09	0.0006	0	0
	E2	0.7841	0.22 ± 0.13	0.1125	0.89 ± 0.15	0.0003	2.79 ± 0.79	0.0031	0.43 ± 0.10	0.0007	0	0

*Ver *cyclins: coelenterates, lower and higher chordates	A1	0.1317	0.50 ± 0.12	0.0008	0.36 ± 0.23	0.1267	3.99 ± 2.25	0.0940	0.39 ± 0.13	0.0082	0	1
	A2	0.6144	0.38 ± 0.4	0	0.78 ± 0.15	0.00007	3.56 ± 0.68	0.00006	0.31 ± 0.04	0	0	0
	B1	0.4895	0.93 ± 0.04	0	0.70 ± 0.20	0.0037	7.88 ± 1.98	0.0016	0.79 ± 0.06	0	0	0
	B2	0.8826	0.87 ± 0.02	0	0.94 ± 0.09	0	5.45 ± 0.47	0	0.70 ± 0.03	0	0	1
	B3	**0.7127**	**0.80 ± 0.15**	**0.0004**	**0.84 ± 0.17**	**0.0006**	**2.23 ± 0.5**	**0.0012**	**0.59 ± 0.13**	**0.0011**	**0**	**1**
	D1	0.7631	0.32 ± 0.05	0.00005	0.87 ± 0.13	0.00002	2.35 ± 0.42	0.00007	0.30 ± 0.04	0.00001	0	1
	D2	0.0208	0.38 ± 0.05	0.000003	0.14 ± 0.26	0.5943	41.0 ± 138	0.771	0.39 ± 0.06	0	0	0
	D3	0.4942	0.36 ± 0.08	0.0019	0.70 ± 0.24	0.0158	3.15 ± 1.11	0.0197	0.34 ± 0.07	0.0011	0	0
	E1	0.6326	0.44 ± 0.07	0.00002	0.80 ± 0.15	0.00008	2.70 ± 0.51	0.00008	0.37 ± 0.06	0	0	0
	E2	0.6198	0.46 ± 0.09	0.00007	0.79 ± 0.15	0.00006	2.46 ± 0.46	0.00005	0.38 ± 0.07	0.00002	0	0

Fungal cyclins	CLB3-4	**0.6515**	**0.69 ± 0.24**	**0.0229**	**0.81 ± 0.22**	**0.0085**	**1.95 ± 0.59**	**0.0132**	**0.53 ± 0.18**	**0.0190**	**1**	**1**
	CDC13	0.1984	1.01 ± 0.13	0.00003	0.45 ± 0.30	0.1698	6.41 ± 4.40	0.1784	0.84 ± 0.21	0.0032	0	0

* Significant δ ≤ 1 and β > 0, evidence for punctual evolution.

** Significant β, node density artefact present; δ < 1 and significant β, evidence for punctual evolution.

Cyclin groups under impact of punctual evolution are in bold.

Comparative embryology provides evidence that the vertebrate embryogenesis was conserved, with similarity more pronounced at early embryogenesis. This is consistent with paleontological evidence: the ecological specialization was narrow in the early vertebrates, represented by relatively monomorphic group of relatively simply organized benthic animals with small body size [96,104]. The vertebrates started to flourish quite late, after the appearance of jaws (allowing to diversify food), and that of two paired limbs providing high mobility [104]. In this way, these two aromorphoses (consequential adaptive changes) preadapted vertebrates to increase in body size and to their eurybiotic nature. The simply organized ancestors of vertebrates widened their tissue repertoire by making their ancestral differentiation mechanisms more elaborate. It should be noted that the mature differentiated somatic cell functions mainly during the G1 cell cycle phase. It of relevant that cyclin D1 together with their paralogs controls precisely G1 phase; moreover, increase in D1 concentration compared with D2 and D3 concentrations is due to the early differentiation of vertebrate germ cells along the neuroectodermal pathway [105]. It may be inferred that punctual evolution of cyclin D1 (Table 3) conforms to the evolution of ectoderm derivatives (neuroectoderm); the evolution of the derivatives of endo- and mesoderm result from the balance between the concentrations of all the tree paralogs and the more gradual evolutionary pattern of cyclins D2 and D3 [105]. The scenario was quite different for invertebrates. Their characteristic features, even within a taxonomic type (or phylum), were wide diversity of embryogenesis, deep metamorphosis (up to holometabolous development and hypermetamorphosis) and stenobiotic ecology. It would seem that for this reason that punctual evolution in invertebrates concerned cyclins A and, possibly cyclins B (Table 3), those controlling the S→G2→M cell cycle transitions, i.e. the most conserved in terms of duration of the cell cycle phases.

Fungal organisms fell into a separate group. Fungi are reducers (decomposers) and/or facultative parasites. Their thallus is organized as syncytium, i.e. nucleus division and the formation of new cell might have been independent of each other, temporally distinct events regulated by major and auxiliary cyclins and auxiliary kinases and/or modifications of major kinases [106-112]. This means that evolution of complexity of fungi should have been nearly neutral with regard to cyclins. This implies the gradual evolution we detected for fungal CDC13. However, events like polyploidization [113-115] or megaduplication [116], might have contributed largely to fungal speciation. This evolutionary pattern is punctuational. In fact, functional specialization in paralogous genes changed during evolution [116]. Ultimately, the newly arisen fungal species (including polyploid species) formed an ecosystem with ancestors [113-115,117,118]. A good example is the punctual evolution of cyclins CLB3-4 in Ascomycota.

It followed that ecological and physiological features of animal and fungal species, which were closely related to the total rate of mutational replacement accumulation, presumably had a considerable impact on cyclin evolution. This conclusion made acute the question of the nature of fixed mutational replacements in different taxa. To examine it closer, we analyzed the rates of amino acid replacements in cyclin families.

### Atypical replacements in evolution of different cyclin families

The first step was to define the periods of evolutionary history, when amino acid changes in cyclins was most dramatic. For this purpose, we performed Markov simulation of protein evolution taking into consideration all the known natural features of cyclin evolution (see Materials and Methods section). As a result, we identified atypical amino acid replacements in certain phylogenetic tree branches for A-, B-, D-, E-type cyclins.

#### Distinguishing features of the evolution of animal A-, B-, D- and E-type cyclins

In vertebrates, accumulation of atypical replacements provided evidence that accelerated evolution in protein function enfolded mainly during cyclin duplication or after it, not in all paralogs, however (Figure 1; Figure 2). Thus, after the split of cyclin A in the common vertebrate ancestor, atypical replacements started to accumulate only in paralog A1. Then, they were found to accumulate faster in both A1 and A2 paralogs, but in different taxa. To illustrate, at the level of the common ancestor of birds and mammals (this corresponded to the emergence of oviparity in the early reptiles), atypical replacements conversely accumulated in A2, while they did not accumulate in A1 (Figure 1A).

The pattern for cyclins D was somewhat more complicated (Figure 1B). A brief increase in the accumulation rates of atypical replacements accompanied diversification of cyclin D1 and the ancestor common to cyclins D2 and D3, then cyclin D2 in particular continued to accumulate atypical replacements; by contrast, cyclin D1 had atypical replacements only at the time of tetrapod rise. Interestingly, the diversification of cyclins D2 and D3 was not associated with accumulation of atypical replacements, but after that replacements of this kind intensely accumulated in paralog D2 in the different vertebrate clades. It is also of interest that against the background of multiple fixations of atypical replacements in paralogs D2 and D1, such events were not observed for D3 evolution.

The evolution of vertebrate cyclins E, at the diversification level of cyclin E paralogs, was obviously similar to that of cyclins D within the D2 and D3 paralog groups (compare Figure 2B and 1B). Diversification of cyclins E1 and E2 was not associated with fixation of atypical replacements, for example. Quite the reverse was observed for the evolutionary scenario within cyclins E1 and E2. They evolved specifically compared with cyclin D paralogs. In this light, of interest were the fixation events of atypical replacements in paralogs E1 and E2 during the rise of mammals, when birds and mammals diverged.

An important pattern became apparent when surveying the evolution of animal B cyclins (Figure 2A). Interestingly, this pattern is peculiar to the evolution of paralog groups of animal cyclins B1 and B2. It is of relevance that B1 is a major and B2 is an auxiliary cyclin [119]. In fact, cyclin B2-null mice develop normally and are fertile whereas cyclin B1-null mice died as embryos [119]. It is also known that cyclin B1 was expressed in interphase in two fractions, free cytoplasmic and membrane-bound, while cyclin B2 was expressed only in membrane-bound form; for this reason, cyclin B1 virtually compensated the absence of cyclin B2, but not vice versa [119]. An important point was that atypical amino acid replacements were distinguishing features at the divergence time of coelenterates and bilaterians. This observation suggested that the animal cyclin B gene possibly acquired multiple functions at this step of evolution.

Analysis of the data in Figure 2A revealed other taxon-specific events of accumulation of atypical replacements. Thus, in invertebrate cyclin B3, acceleration of fixation rate of atypical replacements was detected at the time dipterans and hymenopterans appeared; as for vertebrate cyclin B3, the acceleration became conspicuous when mammals appeared. It is pertinent to note that cyclin B3 is more important for regulation of meiosis than mitosis. In cyclin B3 mutant mice and nematodes, sex cell differentiation was disrupted [84,102,103]. Consequently, it may be suggested that during the divergence time of dipterans and hymenopterans, also of mammals, there occurred considerable changes in meiotic control by B3 cyclins. Cyclins B3 stand aloof from the rest, being relatively conserved in remote vertebrate and invertebrate taxa and containing motifs characteristic of both cyclins B and A [84,102,103,120]. Hence, the coupled fixation of atypical amino acid replacements in cyclins B3, A and E at the formation step of hymenopterans appeared of interest.

#### General features of the evolution of animal A-, B-, D- and E-type cyclins

**Figure 3:**
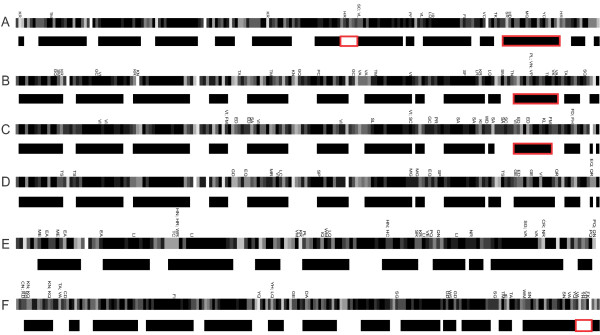
**Schematic representation of mutation distribution in gapless alignment of cyclins**. Cyclins: A, animal A; B, animal B; C, fungal B; D, animal B3; E, animal D; F, animal E. Atypical replacements are indicated above each rectangle in Figures A through E. The upper rectangle in Figures A through E represents the position of amino acids in protein globule sample by color transitions from black, inner amino acids, to white, those on the surface. The lower rectangle in Figures A through E represents the secondary structure in protein sample: black, helical structure, white, turns. Fringes of secondary structure are colored depending on whether the number of all replacements is significantly greater (red) than expected, or as expected (white).

**Figure 4:**
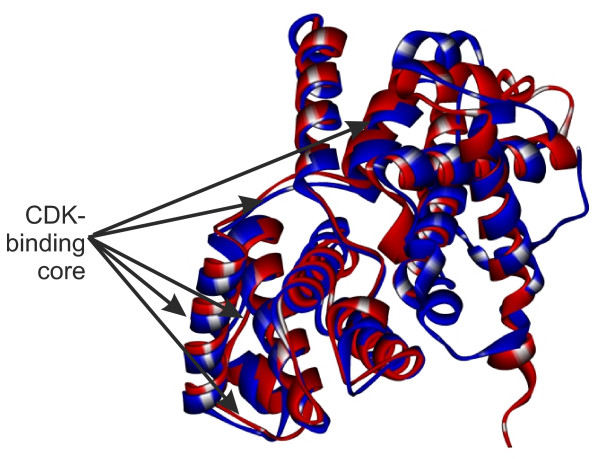
**Alignment of tertiary structures of human cyclin D1 and A1 protein sequences**. Sequences are colored as follows: cyclin D1, red; cyclin A1, blue. Arrows indicate regions binding to CDK.

Thus, the fixation patterns of atypical amino acid replacements in the vertebrate cyclins are specific. However, fragments of these patterns may be similar in inner branches. These particular branches may represent the major aromorphoses (the consequential evolutionary changes), for example: (1) the rise of higher vertebrates, i.e. of the common ancestors of fishes and tetrapods, atypical amino acid replacements were noted for B1- and B2-cyclins (Figure 2A), and A1-cyclins (Figure 1A), D1-cyclins and the common ancestor of D2- and D3-cyclins (Figure 1B); (2) the rise of the tetrapod superclass was marked by accelerated evolution of D2- and D1-cyclins (Figure 1B); (3) the rise of amniotes, the oviparous vertebrates, the common ancestor of birds, reptiles and mammals characterized by atypical replacements in A2-cyclins (Figure 1A); (4) the formation of the common mammalian ancestors observed as accelerated evolution of E2- and D2- cyclins (Figure 1B and 2B); and finally (5) placental viviparity was presumably related to the accelerated evolution of the A1-, E1-, B2- and D2 cyclins (Figure 1; Figure 2). Importantly, two events only were highlighted for the fixation of atypical amino acid replacements in all studied cyclin families: the rise of higher vertebrates (ancestors of fishes and tetrapods) and of placental mammals. The two events possibly needed major developmental changes: in the case of the ancestors of fishes and tetrapods, the formation of jaws and of paired limbs triggered the evolution of all the organismal systems [121,122]; the rise of placental mammals (complete viviparity) required the reorganization of early embryogenesis [123-126].

Also, in vertebrates, atypical amino acid replacements evidenced that accelerated evolution proceeded predominantly during paralog duplication or after it. A model for evolution of gene function through gene duplication was pertinent at this point: it suggested that an ancestral gene had several functions [127]. Clearly, after duplication of a multifunctional gene, the functions of the ancestral gene could evolve independently in the duplicated gene copies, thereby increasing adaptive flexibility [127].

It is of interest that relays were revealed for a number of vertebrate cyclin paralogs. To illustrate, atypical evolution of A1-paralogs was first detected in the lineage of the subtree that corresponed to the fish and tetrapod ancestors, then in the lineage occupied by the common avian, reptilian and mammalian ancestors accelerated evolution in A1-paralogs was substituted by the one for A2-paralogs (Figure 1A). Evolution rate was greater in E2-paralogs of oviparous taxa (birds, reptiles and ancestors of mammals) and in E1-paralogs in placental mammals (Figure 2B).

The reverse was observed for invertebrates. In arthropods, nematodes and echinodermates, the fixation of atypical amino acid replacements was rarely brought into relation with deep evolutionary transitions (aromorphoses). Only the evolution of B-cyclins in the common Metazoa ancestors and ascending to the tip in the specific Coelenterates group (Figure 2A) could be related to aromorphosis (the appearance of true multicellularity). For A- and E-cyclins, atypical amino acid replacements were detected in the lineage of the evolutionary young taxon Hymenoptera; no relations were traced for D-cyclins (Figure 1; Figure 2).

#### Evolutionary features of fungal cyclins

A somewhat different pattern was peculiar to the evolving fungal cyclins (Figure 2A). Accelerated accumulation of atypical replacements was characteristic of just one of the duplicated cyclins, first of only CLB5-6, then of only CLB1-2 and, ultimately, of a distinct subgroup of cyclins within paralog group CDC13. According to Ohno's duplication model, after duplication of a gene performing a single function, one gene copy became subject to directional selection, this led to a dramatic change in its function [128]. In the great majority of cases, one of the gene copies lost its function in the long run under the effect of degenerative mutations. However, both copies may be retained as an outcome of chance acquirement of an important novel function by one of the copies. Of relevance here is the demonstration that in yeast selection against duplicated gene copies was enhanced, while gene copies retained during evolution fulfilled functions different from ancestral [129]. Our current data allowed us to extend Teichmann's (2004) conclusion to the evolution of fungal B cyclins [129]. This appeared reasonable because the distribution pattern of atypical replacements in the fungal phylogenetic tree was consistent with Ohno's neofunctionalization model [128].

Taken together, our current data suggest that cyclin evolution contributed slightly to increasing complexity of invertebrate and fungal organisms. This is supported by the fact that, in contrast to vertebrates, fixation of atypical replacements did not concern earliest evolution (branches at the roots) in invertebrates (Figure 1; Figure 2). Stating it otherwise, all the cases of accelerated evolution of cyclins in invertebrates are located in the terminal branches of phylogenetic tree, thereby providing evidence that events were relatively recent. To the contrary, in the vertebrate lineage (Figure 1; Figure 2), evolution of the four families of major cyclins (except for B3) turned out to be, in one way or another, related to progressive evolution from the emergence of chordates at the time of the Cambrian explosion to at least the divergence of orders. Moreover, for a number of paralogs, progressive evolution correlated in phylogenetic tree with branches corresponding to sharp increases in the number of cell types [96].

It is now evident that accumulation of atypical amino acid replacements during evolution of animal and fungal cyclins fits well into the model for increasing complexity of multicellular organisms through (i) sharp evolutionary aromorphic changes, particularly during vertebrate evolution, related to change in habitats and (ii) gradual differentiation of new functions and separation of the manifold old ones of duplicated genes during evolution of animal and fungi. With all this in mind, the location of the mutational replacements in the different structural-functional cyclin domains posed questions worth considering. We hoped that answers will become clear when the amino acid replacement rates in each secondary structure under study will be known.

### Analysis of replacement rates during the evolution of different cyclin structural elements

#### Positioning of atypical amino acid replacements in the tertiary structure of cyclins

The obvious question was: How atypical amino acid replacenments, which, are, as a rule, rare evolutionary events, may affect the structure of cyclin molecules? The distribution of solvent accessibility values for atypical amino acid replacement positions in gapless alignments showed skewness in favor of either subsurface or surface positions in globule (Table 4). The deviation from symmetry is clear-cut, with clustering above the median in A, B3 and E cyclin types (Table 4). It was also shown that atypical replacements formed local densities on gapless alignments (Figure 3) and that they were predominantly fixed either on the surface or the subsurface regions of the protein globule or where the surface and the buried regions alternated (surface representations are available in Additional file 7).

**Table 4:** Comparison of the accessible surface area (ASA) values of atypical amino acid replacement sites with those of all sites in gapless alignments

	Skewness of atypical replacement samples to positions in the protein globule					
	
	Interior positions in globule (*p*)*			Surface positions in globule (*p*)*		
	
Cyclin families	DSSP ASA values	10 ASA classes	3 ASA classes	DSSP ASA values	10 ASA classes	3 ASA classes
A animal	0.9966	0.9957	0.9906	**0.0034**	**0.0043**	*0.0095*
B animal	0.9570	0.9772	0.9678	0.0432	*0.0230*	0.0324
B fungal	0.6029	0.7785	0.7500	0.3983	0.2224	0.2512
B3 animal	0.9914	0.9975	0.9827	*0.0087*	**0.0025**	*0.0175*
D animal	0.9713	0.9200	0.8835	0.0289	0.0805	0.1173
E animal	0.9995	0.9999	0.9999	**0.0005**	**0.0001**	**0.0001**

*Distribution skewness was estimated using one-tailed Mann-Whitney's test.

*p *≤ 0.005 is in bold, 0.005 <*p *≤ 0.025 is in italics; Bonferonni's correction for simultaneous testing of two statistical hypotheses based on the same dataset: accessible surface area and atypical amino acid replacements positions.

It is known that amino acid replacements in the protein interior affect integral protein characteristics (globule stability and conformation). Surface and subsurface replacements were related to the evolution of distinct active sites (sites of protein-protein interaction or protein-ligand binding) [130]. The decrease in atypical replacement frequency in the interior and their enhanced fixation in the (sub)surface regions was evidence that the cyclin globule conformation was, on the whole, subject to stabilizing selection, which served as a background for local driving evolution of distinct active sites (seen as densities of atypical replacements in gapless alignments (Figure 3)). As known, cyclins are regulatory subunits, which bind, activate and provide substrate specificity of their catalytic partners CDKs [2,5,6]. Fulfilling the function of cell cycle regulators, through control of CDKs substrate specificity, cyclins form short-living complexes with different effector proteins, belonging to transcriptional and co-transcriptional factors, replication factors, signal cascade proteins.

**Figure 5:**
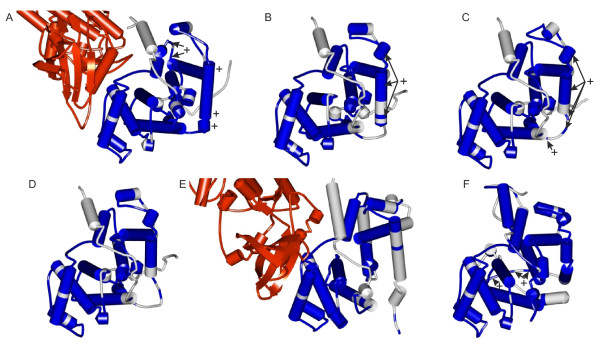
**Three-dimensional structures of cyclins showing the location of conserved and variable secondary structure elements**. A, human cyclin A1-CDK2 complex; B, human B1 cyclin; C, yeast B1 cyclin; D human B3 cyclin; E human cyclin D1-CDK4 complex; F, human E1 cyclin. Secondary structures: replacement number significantly greater than expected is marked "+". CDK molecules are in red; cyclin regions within gapless alignment are in blue.

The statistics for positioning of atypical amino acid replacements is in agreement with the comparative data on cyclin tertiary structures. Figure 4 shows the alignment of protein 3D structures of human cyclins D1 and A2 (RMSD of the structural alignment was 1.9 A, the Z-score = 7). Clearly, most of the perfectly aligned residues consisted of highly conserved α-helices binding to CDKs and formed the internal protein skeleton, whereas the weakly conserved regions comprised the smallest part of the alignment. These regions included the external β-layers, short terminal fragments of the α-helices and loops linking together conserved α-helical regions. The atypical replacements fell, as a rule, in the external α-helical termini and in loops (Figure 3; Figure 5; Additional file 7). The interior regions, usually free of atypical replacements (Figure 3; Figure 5; Additional file 7), corresponded to the central regions of the α-helices.

#### Positioning features of all the amino acid replacements in the tertiary structure of cyclins

The next step was to define the most conserved and the most variable secondary structure elements of the cyclin protein molecule. Using the approach described in the Materials and Methods section, we calculated the total number of amino acid replacements fixed in each element of the protein secondary structure. The secondary structure element, whose number of amino acid replacements was significantly greater than expected, in the case of even distribution of replacements along the sequence, are shown in Figure 3, Figure 5 and Additional file 6.

Analysis of the data in Figure 3 and 5 and Additional file 6 revealed features unique to almost all cyclins (except cyclin D), namely the presence of highly variable regions on the protein surface opposite to the nearly conserved protein region interacting with CDKs. These evolutionary variable sites may conceivably be the acceptors for interaction with other regulator proteins. It is of interest that cyclin D1 has a CDK-independent role as co-activator or co-repressor of tissue-specific transcription factors [e.g. [131-133]]. The results of studies on E-type cyclins also suggested their kinase-independent function. The mutant cyclin E is illustrative: although unable to activate its Cdk2 partners, it can provide the G1/S transition [134]. In the cell, cyclins D and E, in particular, are integrators of signals from extracellular growth factors, thereby defining their crucial role in cell differentiation. Inferences can be made from the results Bryja et al [105] obtained with a model for mammalian cell culture: pluripotent cells prevailed in the cyclin A-CDK2-p27 complex, cyclin E affected proliferation rate and prepared cells to differentiation; the cyclin D2/D3-CDK4-p27 complex prevailed among undifferentiated cells, cyclin D3 elevation in cytoplasm made differentiation follow the endodermal pathway, prevalence of the cyclin D1/D2-CDK4-p27 complex drove differentiation along the neuroectodermal pathway. Replacements considerably different in amino acid properties in the external protein regions can cause the appearance, the disappearance of binding sites in cyclins, or alter them through changes both in surface regions themselves and in the corresponding mutual disposition of internal α-helices and the angles between them (see Figure 3). This suggested that evolutionary changes in external regions of cyclin proteins, animal D, E and B3 in particular, led to changes in the number and properties of cyclin-signal protein binding sites. In fact, estimates of the number of protein-protein interactions for cyclins in different taxa disclosed that it tended to increase with organism complexity (Additional file 8) [135-137]. Taken together, all these facts allow us to explain why cyclin families evolved mainly in the surface and subsurface regions that are far from the centre of CDK binding.

Thus, analysis of the rates of evolutionary reorganization in different regions of cyclin tertiary structure revealed the mechanism possibly underlying the flexibility of cyclin function. This flexibility is provided by very consequential reorganization of subsurface regions remote from interaction sites with CDK in animal and fungal cyclins, also by functional differentiation of paralogous cyclins (including the binding site for CDK) formed during animal evolution. This is the tentative explanation we offer for the differences in the structural-functional evolution between different cyclin families revealed here.

The results for analysis of phylogenetic trees and amino acid replacements may reflect two important features of cyclin evolution: at the organismal level, the evolutionary modes of these proteins differed among taxonomic groups; at the molecular level, the accumulated amino acid replacements possibly provided changes in the interactions of cyclins with other proteins in the cell.

## Conclusions

We studied the molecular evolution of animal and fungal cyclins belonging to the A, B, D and E families. Phylogenetic trees for cyclin protein sequences were constructed, the fixation rates of amino acid replacements were estimated, sets of atypical amino acid replacements, the hallmark features of these cyclins, were identified. We also analyzed the relationship between the molecular evolutionary features of distinct cyclin regions and their structural characteristics in the protein globule. The salient findings were: (1) atypical amino acid replacements were due to aromorphic changes in vertebrates and to gene duplication in animals and fungi during evolution, this was consistent with the model for increasing complexity of multicellular life through changes in habitat, emergence of novel and diversification of old functions in duplicated multifunctional genes; (2) evolutionary flexibility of cyclin functions may be provided by consequential reorganization of surface and subsurface regions remote from CDK interaction sites in animal and fungal cyclins, also by functional differentiation of paralogous cyclins (including CDK binding sites) formed in animal evolution. Cyclin reorganization rates during evolution might have been considerably affected by ecology and physiology of animal and fungi.

Integrating facts and considerations, it may be concluded that the functional roles of cyclins remained interrelated through time at all levels from the molecule-cell-whole organism.

## Authors' contributions

KVG performed all analyses, designed and coordinated the study, suggested the causes of atypical amino acid replacements. VVS suggested the idea that species ecology and protein evolution are related, participated in results discussion. IIT partially participated in writing of Background section. DAA contributed to methodology of the study and participated in results discussion. NAK initiated the study, participated in its coordination. KVG, VVS and DAA contributed significantly to the writing of this paper. All authors read and approved the manuscript.

## Supplementary Material

Additional file 1 **The packed archive contains files with multiple alignments of A-, B-, D-, and E- cyclin protein sequences in FASTA format**. Click here for file

Additional file 2 **The packed archive contains files with gapless multiple alignments of A-, B-, D-, and E- cyclin proteins with reconstructed ancestors in FASTA format**. Click here for file

Additional file 3 **The packed archive contains FASTA-like text files**. Each text file contains space delimited numbers representing reconstruction probabilities of ancestral amino acids in internal tree nodes of A-, B-, D-, and E- cyclin phylogenetic trees for each ancestral sequence. Click here for file

Additional file 4 **The packed archive contains amino acid replacement matrices (in PAML format) for A-, B-, D-, and E- cyclin protein families**. Each matrix file contains additional information for INDELIBLE simulations: the proportion of invariable sites; the shape parameter for the gamma distribution; the number of categories for use in the discrete gamma approximation. Click here for file

Additional file 5 **The packed archive contains MS Excel 2007 files**. Each file represents the data on expected and observed number of amino acid replacements on each branch of the A-, B-, D-, and E- cyclin protein trees with probabilities ***p ***of its observation according to the stationary and homogeneous Markov model of protein evolution. These Excel files also contain the phylogenetic trees with ancestral node labels. Click here for file

Additional file 6 **The packed archive contains MS Excel 2007 files**. Each file represents the following data: (1) the expected and observed numbers of amino acid replacements for each secondary structure element of animal A-, B-, B3-, D- and E-cyclins and fungal B-cyclins; (2) the probabilities ***p ***of its observation under the assumption that fixed replacements are distributed evenly in the protein. Click here for file

Additional file 7 The packed archive contains Accelrys Discovery Studio *msv files. Each file contains 3D protein structure with mapped sites containing atypical amino acid replacements (in blue). Click here for file

Additional file 8 **MS Excel 2007 file contains the number of protein-protein interactions for different paralogs of the A-, B- D-, and E- cyclin proteins according to data deposited in the I2D (Version 1.72), HPRD (Release 8) and DroID (Version 5.0) databases**. Click here for file

## References

[B1] Johnson D Walker C Cyclins and cell cycle checkpoints Annu Rev Pharmacol Toxicol 1999 39 295 312 10.1146/annurev.pharmtox.39.1.295 10331086

[B2] Murray AW Recycling the cell cycle: cyclins revisited Cell 2004 116 221 234 10.1016/S0092-8674(03)01080-8 14744433

[B3] van den Heuvel S The C. Elegans Research Community Cell-cycle regulation WormBook 2005 http://www.wormbook.org

[B4] Evans T Rosenthal ET Youngblom J Distel D Hunt T Cyclin: a protein specified by maternal mRNA in sea urchin eggs that is destroyed at each cleavage division Cell 1983 33 389 396 10.1016/0092-8674(83)90420-8 6134587

[B5] Sherr CJ Roberts JM Living with or without cyclins and cyclin-dependent kinases Genes Dev 2004 18 2699 2711 10.1101/gad.1256504 15545627

[B6] Bloom J Cross FR Multiple levels of cyclin specificity in cell-cycle control Nat Rev Mol Cell Biol 2007 8 149 160 10.1038/nrm2105 17245415

[B7] Coudreuse D Nurse P Driving the cell cycle with a minimal CDK control network Nature 2010 468 1074 1079 10.1038/nature09543 21179163

[B8] Fisher DL Nurse P A single fission yeast mitotic cyclin B p34cdc2 kinase promotes both S-phase and mitosis in the absence of G1 cyclins EMBO J 1996 15 850 860 8631306 PMC450283

[B9] Nurse P Universal control mechanism regulating onset of M-phase Nature 1990 344 503 508 10.1038/344503a0 2138713

[B10] Morgan DO Cyclin-dependent kinases: engines, clocks, and microprocessors Annu Rev Cell Dev Biol 1997 13 261 291 10.1146/annurev.cellbio.13.1.261 9442875

[B11] Wang G Kong H Sun Y Zhang X Zhang W Altman N DePamphilis CW Ma H Genome-wide analysis of the cyclin family in Arabidopsis and comparative phylogenetic analysis of plant cyclin-like proteins Plant Physiol 2004 135 1084 1099 10.1104/pp.104.040436 15208425 PMC514142

[B12] La H Li J Ji Z Cheng Y Li X Jiang S Venkatesh PN Ramachandran S Genome-wide analysis of cyclin family in rice (Oryza Sativa L.) Mol Genet Genomics 2006 275 374 386 10.1007/s00438-005-0093-5 16435118

[B13] Hu X Cheng X Jiang H Zhu S Cheng B Xiang Y Genome-wide analysis of cyclins in maize (Zea mays) Genet Mol Res 2010 9 1490 1503 10.4238/vol9-3gmr861 20690081

[B14] Robbens S Khadaroo B Camasses A Derelle E Ferraz C Inze D Van de Peer Y Moreau H Genome-wide analysis of core cell cycle genes in the unicellular green alga Ostreococcus tauri Mol Biol Evol 2005 22 589 597 15537805 10.1093/molbev/msi044

[B15] Huysman MJ Martens C Vandepoele K Gillard J Rayko E Heijde M Bowler C Inze D Van de Peer Y De Veylder L Vyverman W Genome-wide analysis of the diatom cell cycle unveils a novel type of cyclins involved in environmental signaling Genome Biol 2010 11 R17 10.1186/gb-2010-11-2-r17 20146805 PMC2872877

[B16] Menges M Pavesi G Morandini P Bogre L Murray JA Genomic organization and evolutionary conservation of plant D-type cyclins Plant Physiol 2007 145 1558 1576 10.1104/pp.107.104901 17951462 PMC2151690

[B17] Nieduszynski CA Murray J Carrington M Whole-genome analysis of animal A- and B-type cyclins Genome Biol 2002 3 RESEARCH0070 12537559 10.1186/gb-2002-3-12-research0070 PMC151172

[B18] Li H Coghlan A Ruan J Coin LJ Heriche JK Osmotherly L Li R Liu T Zhang Z Bolund L Wong GK Zheng W Dehal P Wang J Durbin R TreeFam: a curated database of phylogenetic trees of animal gene families Nucleic Acids Res 2006 34 D572 D780 10.1093/nar/gkj118 16381935 PMC1347480

[B19] Suneetha DRS Babu PA Joshua PV The phylogenetic analysis of animal and plant D-type cyclins Trends in Bioinformatics 2008 1 25 32 10.3923/tb.2008.25.32

[B20] Schurko AM Logsdon JM Jr Eads BD Meiosis genes in Daphnia pulex and the role of parthenogenesis in genome evolution BMC Evol Biol 2009 9 78 10.1186/1471-2148-9-78 19383157 PMC2680839

[B21] Drummond DA Bloom JD Adami C Wilke CO Arnold FH Why highly expressed proteins evolve slowly Proc Natl Acad Sci USA 2005 102 14338 14343 10.1073/pnas.0504070102 16176987 PMC1242296

[B22] Drummond DA Wilke CO Mistranslation-induced protein misfolding as a dominant constraint on coding-sequence evolution Cell 2008 134 341 352 10.1016/j.cell.2008.05.042 18662548 PMC2696314

[B23] Drummond DA Wilke CO The evolutionary consequences of erroneous protein synthesis Nat Rev Genet 2009 10 715 724 10.1038/nrg2662 19763154 PMC2764353

[B24] Kanehisa M Goto S Furumichi M Tanabe M Hirakawa M KEGG for representation and analysis of molecular networks involving diseases and drugs Nucleic Acids Res 2010 38 D355 D360 10.1093/nar/gkp896 19880382 PMC2808910

[B25] Kanehisa M Araki M Goto S Hattori M Hirakawa M Itoh M Katayama T Kawashima S Okuda S Tokimatsu T Yamanishi Y KEGG for linking genomes to life and the environment Nucleic Acids Res 2008 36 D480 D484 18077471 10.1093/nar/gkm882 PMC2238879

[B26] Pei J Tang M Grishin NV PROMALS3D web server for accurate multiple protein sequence and structure alignments Nucleic Acids Res 2008 36 W30 W34 10.1093/nar/gkn322 18503087 PMC2447800

[B27] Pei J Kim BH Grishin NV PROMALS3D: a tool for multiple protein sequence and structure alignments Nucleic Acids Res 2008 36 2295 2300 10.1093/nar/gkn072 18287115 PMC2367709

[B28] Buckley TR Model misspecification and probabilistic tests of topology: evidence from empirical data sets Syst Biol 2002 51 509 523 10.1080/10635150290069922 12079647

[B29] Keane TM Creevey CJ Pentony MM Naughton TJ Mclnerney JO Assessment of methods for amino acid matrix selection and their use on empirical data shows that ad hoc assumptions for choice of matrix are not justified BMC Evol Biol 2006 6 29 10.1186/1471-2148-6-29 16563161 PMC1435933

[B30] Arvestad L Efficient methods for estimating amino acid replacement rates J Mol Evol 2006 62 663 673 10.1007/s00239-004-0113-9 16752207

[B31] Guindon S Dufayard J-F Lefort V Anisimova M Hordijk W Gascuel O New Algorithms and Methods to Estimate Maximum-Likelihood Phylogenies: Assessing the Performance of PhyML 3.0 Syst Biol 2010 59 307 321 10.1093/sysbio/syq010 20525638

[B32] Anisimova M Gascuel O Approximate likelihood-ratio test for branches: A fast, accurate, and powerful alternative Syst Biol 2006 55 539 552 10.1080/10635150600755453 16785212

[B33] Shimodaira H Hasegawa M Multiple Comparisons of Log-Likelihoods with Applications to Phylogenetic Inference Mol Biol Evol 1999 16 1114 1116

[B34] Maddison DR Schulz K-S The Tree of Life Web Project 2007 http://tolweb.org

[B35] Burki F Shalchian-Tabrizi K Minge M Skjaeveland A Nikolaev SI Jakobsen KS Pawlowski J Phylogenomics reshuffles the eukaryotic supergroups PloS One 2007 2 e790 10.1371/journal.pone.0000790 17726520 PMC1949142

[B36] Yoon HS Grant J Tekle YI Wu M Chaon BC Cole JC Logsdon JM Jr Patterson DJ Bhattacharya D Katz LA Broadly sampled multigene trees of eukaryotes BMC Evol Biol 2008 8 14 10.1186/1471-2148-8-14 18205932 PMC2249577

[B37] Hampl V Hug L Leigh JW Dacks JB Lang BF Simpson AG Roger AJ Phylogenomic analyses support the monophyly of Excavata and resolve relationships among eukaryotic "supergroups" Proc Natl Acad Sci USA 2009 106 3859 3864 10.1073/pnas.0807880106 19237557 PMC2656170

[B38] Minge MA Silberman JD Orr RJ Cavalier-Smith T Shalchian-Tabrizi K Burki F Skjaeveland A Jakobsen KS Evolutionary position of breviate amoebae and the primary eukaryote divergence Proc Biol Sci 2009 276 597 604 10.1098/rspb.2008.1358 19004754 PMC2660946

[B39] Fitzpatrick DA Logue ME Stajich JE Butler G A fungal phylogeny based on 42 complete genomes derived from supertree and combined gene analysis BMC Evol Biol 2006 6 99 10.1186/1471-2148-6-99 17121679 PMC1679813

[B40] Wang H Xu Z Gao L Hao B A fungal phylogeny based on 82 complete genomes using the composition vector method BMC Evol Biol 2009 9 195 10.1186/1471-2148-9-195 19664262 PMC3087519

[B41] Dopazo H Dopazo J Genome-scale evidence of the nematode-arthropod clade Genome Biol 2005 6 R41 10.1186/gb-2005-6-5-r41 15892869 PMC1175953

[B42] Irimia M Maeso I Penny D Garcia-Fernandez J Roy SW Rare coding sequence changes are consistent with Ecdysozoa, not Coelomata Mol Biol Evol 2007 24 1604 1607 10.1093/molbev/msm105 17525471

[B43] Philippe H Brinkmann H Martinez P Riutort M Baguna J Acoel flatworms are not platyhelminthes: evidence from phylogenomics PloS One 2007 2 e717 10.1371/journal.pone.0000717 17684563 PMC1933604

[B44] Lartillot N Philippe H Improvement of molecular phylogenetic inference and the phylogeny of Bilateria Philos Trans R Soc Lond B Biol Sci 2008 363 1463 1472 10.1098/rstb.2007.2236 18192187 PMC2615818

[B45] Marletaz F Le Parco Y Careful with understudied phyla: the case of chaetognath BMC Evol Biol 2008 8 251 10.1186/1471-2148-8-251 18798978 PMC2566580

[B46] Helmkampf M Bruchhaus I Hausdorf B Phylogenomic analyses of lophophorates (brachiopods, phoronids and bryozoans) confirm the Lophotrochozoa concept Proc Biol Sci 2008 275 1927 1933 10.1098/rspb.2008.0372 18495619 PMC2593926

[B47] Roy SW Irimia M Rare genomic characters do not support Coelomata: intron loss/gain Mol Biol Evol 2008 25 620 623 10.1093/molbev/msn035 18281272

[B48] Podsiadlowski L Braband A Struck TH von Dohren J Bartolomaeus T Phylogeny and mitochondrial gene order variation in Lophotrochozoa in the light of new mitogenomic data from Nemertea BMC Genomics 2009 10 364 10.1186/1471-2164-10-364 19660126 PMC2728741

[B49] Holland LZ Albalat R Azumi K Benito-Gutierrez E Blow MJ Bronner-Fraser M Brunet F Butts T Candiani S Dishaw LJ Ferrier DE Garcia-Fernandez J Gibson-Brown JJ Gissi C Godzik A Hallbook F Hirose D Hosomichi K Ikuta T Inoko H Kasahara M Kasamatsu J Kawashima T Kimura A Kobayashi M Kozmik Z Kubokawa K Laudet V Litman GW McHardy AC The amphioxus genome illuminates vertebrate origins and cephalochordate biology Genome Res 2008 18 1100 1111 10.1101/gr.073676.107 18562680 PMC2493399

[B50] Swalla BJ Smith AB Deciphering deuterostome phylogeny: molecular, morphological and palaeontological perspectives Philos Trans R Soc Lond B Biol Sci 2008 363 1557 1568 10.1098/rstb.2007.2246 18192178 PMC2615822

[B51] Singh TR Tsagkogeorga G Delsuc F Blanquart S Shenkar N Loya Y Douzery EJ Huchon D Tunicate mitogenomics and phylogenetics: peculiarities of the Herdmania momus mitochondrial genome and support for the new chordate phylogeny BMC Genomics 2009 10 534 10.1186/1471-2164-10-534 19922605 PMC2785839

[B52] Hallstrom BM Kullberg M Nilsson MA Janke A Phylogenomic data analyses provide evidence that Xenarthra and Afrotheria are sister groups Mol Biol Evol 2007 24 2059 2068 10.1093/molbev/msm136 17630282

[B53] Kitazoe Y Kishino H Waddell PJ Nakajima N Okabayashi T Watabe T Okuhara Y Robust time estimation reconciles views of the antiquity of placental mammals PloS One 2007 2 e384 10.1371/journal.pone.0000384 17440620 PMC1849890

[B54] Kjer KM Honeycutt RL Site specific rates of mitochondrial genomes and the phylogeny of eutheria BMC Evol Biol 2007 7 8 10.1186/1471-2148-7-8 17254354 PMC1796853

[B55] Murphy WJ Pringle TH Crider TA Springer MS Miller W Using genomic data to unravel the root of the placental mammal phylogeny Genome Res 2007 17 413 421 10.1101/gr.5918807 17322288 PMC1832088

[B56] Hou ZC Romero R Wildman DE Phylogeny of the Ferungulata (Mammalia: Laurasiatheria) as determined from phylogenomic data Mol Phylogenet Evol 2009 52 660 664 10.1016/j.ympev.2009.05.002 19435603 PMC3539735

[B57] Krauss V Thummler C Georgi F Lehmann J Stadler PF Eisenhardt C Near intron positions are reliable phylogenetic markers: an application to holometabolous insects Mol Biol Evol 2008 25 821 830 10.1093/molbev/msn013 18296416

[B58] Kolaczkowski B Thornton JW A mixed branch length model of heterotachy improves phylogenetic accuracy Mol Biol Evol 2008 25 1054 1066 10.1093/molbev/msn042 18319244 PMC3299401

[B59] Archambault V Buchler NE Wilmes GM Jacobson MD Cross FR Two-faced cyclins with eyes on the targets Cell Cycle 2005 4 125 130 10.4161/cc.4.1.1402 15611618

[B60] Cai W Pei J Grishin NV Reconstruction of ancestral protein sequences and its applications BMC Evol Biol 2004 4 33 10.1186/1471-2148-4-33 15377393 PMC522809

[B61] Whelan S Goldman N A general empirical model of protein evolution derived from multiple protein families using a maximum-likelihood approach Mol Biol Evol 2001 18 691 699 11319253 10.1093/oxfordjournals.molbev.a003851

[B62] Pupko T Pe'er I Hasegawa M Graur D Friedman N A branch-and-bound algorithm for the inference of ancestral amino-acid sequences when the replacement rate varies among sites: Application to the evolution of five gene families Bioinformatics 2002 18 1116 1123 10.1093/bioinformatics/18.8.1116 12176835

[B63] Le SQ Gascuel O An improved general amino acid replacement matrix Mol Biol Evol 2008 25 1307 1320 10.1093/molbev/msn067 18367465

[B64] Yang Z PAML 4: phylogenetic analysis by maximum likelihood Mol Biol Evol 2007 24 1586 1591 10.1093/molbev/msm088 17483113

[B65] Yang Z Kumar S Nei M A new method of inference of ancestral nucleotide and amino acid sequences Genetics 1995 141 1641 1650 8601501 10.1093/genetics/141.4.1641 PMC1206894

[B66] Fletcher W Yang Z INDELible: a flexible simulator of biological sequence evolution Mol Biol Evol 2009 26 1879 1888 10.1093/molbev/msp098 19423664 PMC2712615

[B67] Gunbin KV Afonnikov DA Kolchanov NA Molecular evolution of the hyperthermophilic archaea of the Pyrococcus genus: analysis of adaptation to different environmental conditions BMC Genomics 2009 10 639 10.1186/1471-2164-10-639 20042074 PMC2816203

[B68] Mielke PW Berry KJ Permutation Methods: A Distance Function Approach 2007 2 NY, Springer Science+Business Media

[B69] Kabsch W Sander C Dictionary of protein secondary structure: pattern recognition of hydrogen-bonded and geometrical features Biopolymers 1983 22 2577 2637 10.1002/bip.360221211 6667333

[B70] Berman HM Westbrook J Feng Z Gilliland G Bhat TN Weissig H Shindyalov IN Bourne PE The Protein Data Bank Nucleic Acids Res 2000 28 235 242 10.1093/nar/28.1.235 10592235 PMC102472

[B71] Kelley LA Sternberg MJ Protein structure prediction on the Web: a case study using the Phyre server Nat Protoc 2009 4 363 371 19247286 10.1038/nprot.2009.2

[B72] Shindyalov IN Bourne PE Protein structure alignment by incremental combinatorial extension (CE) of the optimal path Protein Eng 1998 11 739 747 10.1093/protein/11.9.739 9796821

[B73] Ahmad S Gromiha M Fawareh H Sarai A ASAView: database and tool for solvent accessibility representation in proteins BMC Bioinformatics 2004 5 51 10.1186/1471-2105-5-51 15119964 PMC420234

[B74] R Development Core Team R: A language and environment for statistical computing 2010 Vienna, R Foundation for Statistical Computing http://www.R-project.org

[B75] Payne JL Boyer AG Brown JH Finnegan S Kowalewski M Krause RA Jr Lyons SK McClain CR McShea DW Novack-Gottshall PM Smith FA Stempien JA Wang SC Two-phase increase in the maximum size of life over 3.5 billion years reflects biological innovation and environmental opportunity Proc Natl Acad Sci USA 2009 106 24 27 10.1073/pnas.0806314106 19106296 PMC2607246

[B76] Zherikhin VV Ponomarenko AG Rasnitsyn AP Introduction into palaeoentomology 2008 Moscow, KMK Press [in Russian]

[B77] Ricklefs RE Life-history connections to rates of aging in terrestrial vertebrates Proc Natl Acad Sci USA 2010 107 10314 10319 10.1073/pnas.1005862107 20479246 PMC2890449

[B78] Schmidt-Nielson K Scaling: Why is Animal Size so Important? 1984 New York, Cambridge University Press

[B79] Holton TA Pisani D Deep genomic-scale analyses of the metazoa reject Coelomata: evidence from single- and multigene families analyzed under a supertree and supermatrix paradigm Genome Biol Evol 2010 2 310 324 10.1093/gbe/evq016 20624736 PMC2997542

[B80] Sulston JE Schierenberg E White JG Thomson JN The embryonic cell lineage of the nematode Caenorhabditis elegans Dev Biol 1983 100 64 119 10.1016/0012-1606(83)90201-4 6684600

[B81] Aleshin VV Whether variable cleavage of Enoplida (Nematoda) is primitive? Notes to D.A. Voronov article "Comparative embryology of Nematoda and the law of embryologic similarity Zh Obshch Biol 2004 65 74 80 [in Russian] 15032066

[B82] Boxem M Cyclin-dependent kinases in C.elegans Cell Div 2006 1 6 10.1186/1747-1028-1-6 16759361 PMC1482691

[B83] Fujita M Takeshita H Sawa H Cyclin E and CDK2 repress the terminal differentiation of quiescent cells after asymmetric division in C.elegans PloS One 2007 2 e407 10.1371/journal.pone.0000407 17476329 PMC1852333

[B84] van der Voet M Lorson MA Srinivasan DG Bennett KL van den Heuvel S C. Elegans mitotic cyclins have distinct as well as overlapping functions in chromosome segregation Cell Cycle 2009 8 4091 4102 10.4161/cc.8.24.10171 19829076 PMC3614003

[B85] Doonan JH Cell division in Aspergillus J Cell Sci 1992 103 599 611 1336015 10.1242/jcs.103.3.599

[B86] Lew DJ Reed SI Morphogenesis in the yeast cell cycle: regulation by Cdc28 and cyclins J Cell Biol 1993 120 1305 1320 10.1083/jcb.120.6.1305 8449978 PMC2119756

[B87] Fischer R Nuclear movement in filamentous fungi FEMS Microbiol Rev 1999 23 39 68 10.1111/j.1574-6976.1999.tb00391.x 10077853

[B88] Gale C Gerami-Nejad M McClellan M Vandoninck S Longtine MS Berman J Candida albicans Int1p interacts with the septin ring in yeast and hyphal cells Mol Biol Cell 2001 12 3538 3549 11694587 10.1091/mbc.12.11.3538 PMC60274

[B89] Ah Fong AM Judelson HS Cell cycle regulator Cdc14 is expressed during sporulation but not hyphal growth in the fungus-like oomycete Phytophthora infestans Mol Microbiol 2003 50 487 494 10.1046/j.1365-2958.2003.03735.x 14617173

[B90] Wightman R Bates S Amornrrattanapan P Sudbery P In Candida albicans, the Nim1 kinases Gin4 and Hsl1 negatively regulate pseudohypha formation and Gin4 also controls septin organization J Cell Biol 2004 164 581 591 10.1083/jcb.200307176 14769857 PMC2171991

[B91] Sudbery P Morphogenesis of a human fungal pathogen requires septin phosphorylation Dev Cell 2007 13 315 316 10.1016/j.devcel.2007.08.009 17765672

[B92] Egelhofer TA Villen J McCusker D Gygi SP Kellogg DR The septins function in G1 pathways that influence the pattern of cell growth in budding yeast PloS One 2008 3 e2022 10.1371/journal.pone.0002022 18431499 PMC2291192

[B93] Colomina N Ferrezuelo F Verges E Aldea M Gari E Whi3 regulates morphogenesis in budding yeast by enhancing Cdk functions in apical growth Cell Cycle 2009 8 1912 1920 10.4161/cc.8.12.8740 19440046

[B94] Castiglioni Pascon R Pizzirani-Kleiner AA Miller BL The Aspergillus nidulans bncA1 mutation causes defects in the cell division cycle, nuclear movement and developmental morphogenesis Mol Gen Genet 2001 264 546 554 10.1007/s004380000360 11212909

[B95] Wei H Requena N Fischer R The MAPKK kinase SteC regulates rtefact ores morphology and is essential for heterokaryon formation and sexual development in the homothallic fungus Aspergillus nidulans Mol Microbiol 2003 47 1577 1588 10.1046/j.1365-2958.2003.03405.x 12622813

[B96] Hedges SB Blair JE Venturi ML Shoe JL A molecular timescale of eukaryote evolution and the rise of complex multicellular life BMC Evol Biol 2004 4 1 9 10.1186/1471-2148-4-1 15005799 PMC341452

[B97] Elena SF Lenski RE Test of synergistic interactions among deleterious mutations in bacteria Nature 1997 390 395 398 10.1038/37108 9389477

[B98] Elena SF Lenski RE Microbial genetics: Evolution experiments with microorganisms: the dynamics and genetic bases of adaptation Nat Rev Genet 2003 4 457 469 12776215 10.1038/nrg1088

[B99] Pagel M Venditti C Meade A Large punctuational contribution of speciation to evolutionary divergence at the molecular level Science 2006 314 119 121 10.1126/science.1129647 17023657

[B100] Venditti C Meade A Pagel M Detecting the node-density artefact in phylogeny reconstruction Syst Biol 2006 55 637 643 10.1080/10635150600865567 16969939

[B101] Webster AJ Payne RJH Pagel M Molecular Phylogenies Link Rates of Evolution and Speciation Science 2003 301 478 10.1126/science.1083202 12881561

[B102] Nguyen TB Manova K Capodieci P Lindon C Bottega S Wang XY Refik-Rogers J Pines J Wolgemuth DJ Koff A Characterization and expression of mammalian cyclin b3, a prepachytene meiotic cyclin J Biol Chem 2002 277 41960 41969 10.1074/jbc.M203951200 12185076

[B103] Miles DC van den Bergen JA Sinclair AH Western PS Regulation of the female mouse germ cell cycle during entry into meiosis Cell Cycle 2010 9 408 418 10.4161/cc.9.2.10691 20023406

[B104] Carroll RL Vertebrate Paleontology and Evolution 1988 New York, WH Freeman and Company

[B105] Bryja V Pachernik J Vondracek J Soucek K Cajanek L Horvath V Holubcova Z Dvorak P Hampl A Lineage specific composition of cyclin D-CDK4/CDK6-p27 complexes reveals distinct functions of CDK4, CDK6 and individual D-type cyclins in differentiating cells of embryonic origin Cell Prolif 2008 41 875 893 10.1111/j.1365-2184.2008.00556.x 19040567 PMC2659368

[B106] Tasto JJ Morrell JL Gould KL An anillin homologue, Mid2p, acts during fission yeast cytokinesis to organize the septin ring and promote cell separation J Cell Biol 2003 160 1093 1103 10.1083/jcb.200211126 12668659 PMC2172762

[B107] Asano S Park JE Sakchaisri K Yu LR Song S Supavilai P Veenstra TD Lee KS Concerted mechanism of Swe1/Wee1 regulation by multiple kinases in budding yeast EMBO J 2005 24 2194 2204 10.1038/sj.emboj.7600683 15920482 PMC1150880

[B108] Lew DJ Reed SI Morphogenesis in the yeast cell cycle: regulation by Cdc28 and cyclins J Cell Biol 1993 120 1305 1320 10.1083/jcb.120.6.1305 8449978 PMC2119756

[B109] Colomina N Ferrezuelo F Vergés E Aldea M Gari E Whi3 regulates morphogenesis in budding yeast by enhancing Cdk functions in apical growth Cell Cycle 2009 8 1912 1920 10.4161/cc.8.12.8740 19440046

[B110] Longtine MS Theesfeld CL McMillan JN Weaver E Pringle JR Lew DJ Septin-dependent assembly of a cell cycle-regulatory module in Saccharomyces cerevisiae Mol Cell Biol 2000 20 4049 4061 10.1128/MCB.20.11.4049-4061.2000 10805747 PMC85775

[B111] Sudbery P Morphogenesis of a human fungal pathogen requires septin phosphorylation Dev Cell 2007 13 315 316 10.1016/j.devcel.2007.08.009 17765672

[B112] Wightman R Bates S Amornrrattanapan P Sudbery P In Candida albicans, the Nim1 kinases Gin4 and Hsl1 negatively regulate pseudohypha formation and Gin4 also controls septin organization J Cell Biol 2004 164 581 591 10.1083/jcb.200307176 14769857 PMC2171991

[B113] Naumov GI Naumova ES Smith MT de Hoog GS Molecular-genetic diversity of the ascomycetous yeast genus Arthroascus: Arthroascus babjevae sp. Nov., Arthroascus fermentans var. Arxii var. Nov. And geographical populations of Arthroascus schoenii Int J Syst Evol Microbiol 2006 56 1997 2007 10.1099/ijs.0.64301-0 16902043

[B114] Naumova ES Ivannikova IuV Naumov GI Genetic differentiation of the sherry yeasts Saccharomyces cerevisiae Prikl Biokhim Mikrobiol 2005 41 656 661 [in Russian] 16358756

[B115] Naumova ES Naumov GI Masneuf-Pomarede I Aigle M Dubourdieu D Molecular genetic study of introgression between Saccharomyces bayanus and S. Cerevisiae Yeast 2005 22 1099 1115 10.1002/yea.1298 16240458

[B116] Piskur J Origin of duplicated regions in the yeast genomes Trends Genet 2001 17 302 303 10.1016/S0168-9525(01)02308-3 11377778

[B117] Cliften PF Fulton RS Wilson RK Johnston M After the duplication: gene loss and adaptation in Saccharomyces genomes Genetics 2006 172 863 872 16322519 10.1534/genetics.105.048900 PMC1456250

[B118] Kellis M Birren BW Lander ES Proof and evolutionary analysis of ancient genome duplication in the yeast Saccharomyces cerevisiae Nature 2004 428 617 624 10.1038/nature02424 15004568

[B119] Brandeis M Rosewell I Carrington M Crompton T Jacobs MA Kirk J Gannon J Hunt T Cyclin B2-null mice develop normally and are fertile whereas cyclin B1-null mice die in utero Proc Natl Acad Sci USA 1998 95 4344 4349 10.1073/pnas.95.8.4344 9539739 PMC22491

[B120] Gallant P Nigg EA Identification of a novel vertebrate cyclin: cyclin B3 shares properties with both A- and B-type cyclins EMBO J 1994 13 595 605 8313904 10.1002/j.1460-2075.1994.tb06297.x PMC394849

[B121] Romer AS Parsons TS The Vertebrate Body, 6th Edition 1986 Saunders, Philadelphia, Saunders College Publishing

[B122] Shubin N Your Inner Fish 2008 London, Allen Lane

[B123] Gilbert SF Developmental Biology, 7th Edition 2003 Sunderland, Sinauer Associates, Inc

[B124] Cross JC Baczyk D Dobric N Hemberger M Hughes M Simmons DG Yamamoto H Kingdom JC Genes, development and evolution of the placenta Placenta 2003 24 123 130 10.1053/plac.2002.0887 12596737

[B125] Knox K Baker JC Genomic evolution of the placenta using co-option and duplication and divergence Genome Res 2008 18 695 705 10.1101/gr.071407.107 18340042 PMC2336813

[B126] Old LJ Cancer is a somatic cell pregnancy Cancer Immunity 2007 7 19 21 17983204 PMC2935741

[B127] Hughes AL The evolution of functionally novel proteins after gene duplication Proc R Soc Lond Ser B Biol Sci 1994 256 119 124 10.1098/rspb.1994.0058 8029240

[B128] Ohno S Evolution by gene duplication 1970 New York, Springer-Verlag

[B129] Teichmann SA Babu MM Gene regulatory network growth by duplication Nat Genet 2004 36 492 496 10.1038/ng1340 15107850

[B130] Jones S Thornton JM Principles of protein-protein interactions Proc Natl Acad Sci USA 1996 93 13 20 10.1073/pnas.93.1.13 8552589 PMC40170

[B131] Zwijsen RM Wientjens E Klompmaker R van der Sman J Bernards R Michalides RJ CDK-independent activation of estrogen receptor by cyclin D1 Cell 1997 88 405 415 10.1016/S0092-8674(00)81879-6 9039267

[B132] Fu M Rao M Bouras T Wang C Wu K Zhang X Li Z Yao TP Pestell RG Cyclin D1 inhibits peroxisome proliferator-activated receptor gamma-mediated adipogenesis through histone deacetylase recruitment J Biol Chem 2005 280 16934 16941 10.1074/jbc.M500403200 15713663

[B133] Inoue K Sherr CJ Gene expression and cell cycle arrest mediated by transcription factor DMP1 is antagonized by D-type cyclins through a cyclin-dependent-kinase-independent mechanism Mol Cell Biol 1998 18 1590 1600 9488476 10.1128/mcb.18.3.1590 PMC108874

[B134] Geng Y Young-Mi Lee Y-M Welcker M Swanger J Zagozdzon A Winer JD Roberts JM Kaldis P Clurman BE Sicinski P Kinase-Independent Function of Cyclin E Mol Cell 25 127 139 10.1016/j.molcel.2006.11.029 17218276

[B135] Brown KR Jurisica I Online Predicted Human Interaction Database Bioinformatics 2005 21 2076 2082 10.1093/bioinformatics/bti273 15657099

[B136] Keshava Prasad TS Goel R Kandasamy K Keerthikumar S Kumar S Mathivanan S Telikicherla D Raju R Shafreen B Venugopal A Balakrishnan L Marimuthu A Banerjee S Somanathan DS Sebastian A Rani S Ray S Harrys Kishore CJ Kanth S Ahmed M Kashyap MK Mohmood R Ramachandra YL Krishna V Rahiman BA Mohan S Ranganathan P Ramabadran S Chaerkady R Pandey A Human Protein Reference Database--2009 update Nucleic Acids Res 2009 37 D767 D772 10.1093/nar/gkn892 18988627 PMC2686490

[B137] Murali T Pacifico S Yu J Guest S Roberts GG Finley RL Jr DroID 2011: a comprehensive, integrated resource for protein, transcription factor, RNA and gene interactions for Drosophila Nucleic Acids Res 2011 39 D736 D743 10.1093/nar/gkq1092 21036869 PMC3013689

